# Cross-species analysis of hepatic cytochrome P450 and transport protein expression

**DOI:** 10.1007/s00204-020-02939-4

**Published:** 2020-11-04

**Authors:** Helen Hammer, Felix Schmidt, Philip Marx-Stoelting, Oliver Pötz, Albert Braeuning

**Affiliations:** 1 Signatope, Markwiesenstraße 55, 72770 Reutlingen, Germany; 2 Department of Pesticides Safety, German Federal Institute for Risk Assessment, Max-Dohrn-Str. 8-10, 10589 Berlin, Germany; 3 Department of Food Safety, German Federal Institute for Risk Assessment, Max-Dohrn-Str. 8-10, 10589 Berlin, Germany

**Keywords:** ABC transporter, Azole fungicides, Cytochrome P450, Hepatocytes, Humanized mouse models, Nuclear receptors, SLC transporter, TXP, Xenobiotic metabolism

## Abstract

Most drugs and xenobiotics are metabolized in the liver. Amongst others, different cytochrome P450 (CYP) enzymes catalyze the metabolic conversion of foreign compounds, and various transport proteins are engaged in the excretion of metabolites from the hepatocytes. Inter-species and inter-individual differences in the hepatic levels and activities of drug-metabolizing enzymes and transporters result from genetic as well as from environmental factors, and play a decisive role in determining the pharmacokinetic properties of a compound in a given test system. To allow for a meaningful comparison of results from metabolism studies, it is, therefore, of utmost importance to know about the specific metabolic properties of the test systems, especially about the levels of metabolic enzymes such as the CYPs. Using a targeted proteomics approach, we, therefore, compared the hepatic levels of important CYP enzymes and transporters in different experimental systems in vivo and in vitro, namely Wistar rats, C57/Bl6 mice, mice humanized for the two xeno-sensing receptors PXR (pregnane-X-receptor) and CAR (constitutive androstane receptor), mice with human hepatocyte-repopulated livers, human HepaRG hepatocarcinoma cells, primary human hepatocytes, and human liver biopsies. In addition, the effects of xenobiotic inducers of drug metabolism on CYP enzymes and transporters were analyzed in selected systems. This study for the first time presents a comprehensive overview of similarities and differences in important drug metabolism-related proteins among the different experimental models.

**Electronic supplementary material:**

The online version of this article (10.1007/s00204-020-02939-4) contains supplementary material, which is available to authorized users.

## Introduction

Induction of xenobiotic metabolism in mammalian liver is regulated by a set of nuclear receptors among which the aryl hydrocarbon receptor (AHR), the constitutive androstane receptor (CAR) and the pregnane-X-receptor (PXR) are the most prominent ones, as for example reviewed by Honkakoski and Negishi (2000), Kobayashi et al. (2015), and Kohle and Bock (2007). The receptors are activated following exposure to xenobiotics and subsequently induce the expression of several xenobiotic metabolism-related genes, e.g., encoding metabolic enzymes such as cytochrome P450 (CYP) enzymes or transporters capable of exporting xenobiotic substances from cells. Significant inter-species differences exist with respect to receptor activation and enzyme inducibility by chemical compounds, which have to be analyzed in a case-by-case manner to reach sound conclusions on the human relevance of toxic effects observed in the rodent species generally used in the risk assessment of chemicals. While comparative gene expression arrays are one tool to obtain information on species-specific induction of CYP and transporter genes, new targeted proteomics approaches like triple-X-proteomics (TXP) allow for a comprehensive and rapid analysis of specific nuclear receptor targets such as CYPs and transporters at the protein level.

In mammals, CYPs are located at the cytosolic side of the endoplasmic reticulum and the inner membrane of the mitochondria and serve two major functions (Williams et al. 2000). While many CYPs are part of the biosynthesis and metabolism of endogenous substances such as hormones, bile acids and vitamins, the CYP family 1, 2 and 3 members constitute major players in the oxidative metabolism of xenobiotics (Martignoni et al. 2006; Williams et al. 2000). The CYP1A subfamily consists of two highly conserved proteins in human, mouse and rat. CYP1A1 and CYP1A2 recognize planar compounds such as polycyclic aromatic hydrocarbons and arylamines as substrates. The CYP2A subfamily includes three human, three rattine and four murine isoforms. Even though CYPs are classified across species, minor differences in the amino acid sequence of the protein may lead to drastic changes in substrate specificity and catalytic activity. The rodent CYP2A isoforms, for example, catalyze the hydroxylation of steroids, while human CYP2A6 oxidizes substances such as aflatoxin B1 and nicotine, and shows a great substrate overlap with CYP2E1. CYP2C is the most diverse subfamily harboring four human, seven rattine and nine murine enzymes. As for CYP2A, the substrate specificities differ greatly between human and rodent CYP2C isoforms. In addition, the expression of some isoforms is gender dependent in adult rats, e.g., the female- and male-specific isoforms CYP2C12 and Cyp2C13 (Martignoni et al. 2006). The subfamily CYP3A recognizes a very broad range of substrates and is, therefore, very important in drug and xenobiotic metabolism. Humans express four and rats and mice each express six CYP3A isoforms (Martignoni et al. 2006). It was estimated that together, CYPs are involved in the metabolism of 70–80% of all clinically used drugs (Sutton et al. 2010).

Even if the enzymes themselves are conserved, their inducibility by different receptors and across different species is not [e.g., see Graham and Lake (2008) and Karpen (2002)]. Significant species differences are especially discussed for inducers of CAR (Braeuning and Schwarz 2016; Elcombe et al. 2014). To facilitate regulatory decisions on human relevance methods are required that are capable of quantifying the response of nuclear receptor inducers across different species and test systems in a robust and reliable manner.

There are several forms of transport proteins in mammals. With respect to xenobiotic metabolism, a group of active transporters containing an ATP-binding cassette (ABC), thus named ABC transporters, are most prominent (Wilkens 2015). In addition, also solute carrier proteins (SLC) play an important role as molecules facilitating non-active transport through cellular membranes (Lin et al. 2015). These transporters, like the CYPs, have a broad substrate specificity and are grouped into distinct families. In addition, similar to the CYPs, transcriptional induction of transporters by nuclear receptors such as AHR, CAR or PXR is a well-known phenomenon that may also differ between the species (Scotto 2003).

Azole fungicides are widely used in agriculture and in human and veterinary medicine to treat a broad spectrum of fungal disease. From a mechanistic perspective, these substances are designed to inhibit fungal CYP51 (lanosterol-14*α*-demethylase) to block cell membrane synthesis (Georgopapadakou 1998). As a side effect, they may inhibit mammalian CYP enzymes in an unspecific manner, but they are also activators of hepatic nuclear receptors such as CAR, PXR and AHR (Marx-Stoelting et al. 2020). Azole-induced liver effects may exhibit significant species differences: For example, Rieke et al. (2017) showed that hepatic responses to the azole fungicide cyproconazole were remarkably less pronounced in mice with humanized CAR and PXR as compared to wild-type controls, suggesting that the compound was not able to stimulate the receptors from the two species to a comparable degree. Since the ability of some azoles to activate nuclear receptors significantly differs between species [for review see Marx-Stoelting et al. (2020)], this group of compounds was chosen as CYP and transporter inducers in test systems of different origin in the present study. As test systems, human in vitro models (liver biopsies, primary hepatocytes, and HepaRG hepatocarcinoma cells), wild-type mouse and rat liver, a CAR/PXR-humanized mouse model as well as a mouse model with human hepatocyte-repopulated livers were chosen to cover a broad spectrum from laboratory animals to human material. Protein expression of important CYPs and transporters was quantified using mass spectrometry in combination with peptide group-specific enrichment and motif-specific antibodies (TXP antibodies) (Poetz et al. 2009; Weiss et al. 2018). The use of these antibodies enables a fast and reliable quantification of closely related CYP isoforms and transporters at the protein level. This study shows the ability to apply these antibodies in cross-species analyses.

## Materials and methods

### Test compounds and animal diet formulation

Technical grade compounds (i.e., identical quality and purity as used in plant protection products) were obtained directly from the producing companies: cyproconazole (CAS no. 94361-06-9, Batch no. CHF1E00042, Purity 96.8%) was purchased from Syngenta (Basel, Switzerland) and prochloraz (CAS no. 67747-09-5, Batch no. COD-000718, Purity 98.0%) was supplied by BASF (Ludwigshafen, Germany). For animal studies, compounds were mixed into phytoestrogen-free standard rodent chow (R/M-H V155; Ssniff, Soest, Germany) using a solvent-free procedure (Schmidt et al. 2016). Concentration and stability of test substances in the rodent diet was checked as described previously (Heise et al. 2015). Accordingly, the control diet was checked for the absence of pesticides, especially triazole fungicides, to ensure the quality of the negative control (Schmidt et al. 2016). Dose selection was based on the no adverse effect levels (NOAELs) of regulatory studies available from the approval procedures of the individual active substances, as detailed previously (Schmidt et al. 2016). Doses were chosen to equal daily ingestion of an amount of the respective test compound around the NOAELs (rat: 6.4 mg/kg body weight/day cyproconazole, 6 mg/kg body weight/day prochloraz; mouse: 2.2 mg/kg body weight/day cyproconazole, 6 mg/kg body weight/day prochloraz), or around ten times the NOAEL (EFSA 2010; EFSA 2011). For in vitro experiments, the test compounds were dissolved in dimethyl sulfoxide (DMSO).

### Tissue samples

Liver tissue samples of rats, mice, and CAR/PXR-humanized mice were available from previously published studies (Heise et al. 2015; Marx-Stoelting et al. 2017; Schmidt et al. 2016). For experimental details, please refer to the latter publications. In brief, healthy 9-week-old male Wistar rats (Crl/Wi background; Charles River, Sulzfeld, Germany) received either standard diet or one of the test substance-containing diets and filtered tap water ad libitum for 28 days. Similarly, 8-week-old male wild-type mice, as well as transgenic mice with humanized *CAR* and *PXR* (C57/Bl6 background; Taconic, Cologne, Germany), received either standard diet or one of the test substance-containing diets for 28 days. Group size was *n* = 5 animals per group in both studies, with the exception of the vehicle control group for which samples from *n* = 10 animals were available; for test diet formulation please refer to the above paragraph. Animals were checked daily for clinical signs and mortality. Animals were euthanized after 28 days of treatment. Directly after isolation, livers were frozen on dry ice for subsequent molecular analysis. The experiments were conducted with males only, since they were slightly more sensitive according to previous studies from the approval procedures for the respective fungicides.

Samples from male mice with humanized livers were available from Yecuris corporation (Tualatin, Oregon, USA). The immunodeficient, so-called “FRG-KO” mice are deficient in the tyrosine catabolic enzyme fumarylacetoacetate hydrolase, and their livers can be used for repopulation by human hepatocytes (Azuma et al. 2007). According to information from Yecuris, the livers displayed levels of > 90% humanization by hepatocytes from donor HHF13023 (donor 1), a 13-year-old female Caucasian. Liver samples from eight mice were frozen on dry ice for subsequent molecular analysis immediately after killing.

Fresh frozen primary human hepatocytes from three donors (donors 2–4) were obtained from HTCR Services GmbH (Planegg/Martinsried, Germany). Cells from donors A927869, A403202 and A256912 (one male and two female, 30–79 years) were thawed and directly prepared for analysis without cultivation or treatment. Analyses with human primary hepatocytes were performed in technical triplicates. In addition, five human liver biopsies, which have already been analyzed and published previously, are included for comparison (donors 5–9). For experimental details, please see Weiss et al. (2018).

### In vitro samples

Undifferentiated HepaRG cells were obtained from Biopredic International (Saint Grégoire, France). The cells were grown at 37 °C in a humidified atmosphere with 5% CO_2_ in 10-cm-diameter cell culture plates. Initial 2 weeks of cultivation in William’s E medium (Pan-Biotech GmbH, Aidenbach, Germany) containing 10% fetal calf serum (FCS) (PAN-Biotech GmbH, Aidenbach, Germany), 100 U/mL penicillin, 100 µg/mL streptomycin, 0.05% human insulin (all from PAA Laboratories GmbH, Pasching, Austria) and 50 µM hydrocortisone-hemisuccinate (Sigma-Aldrich, Taufkirchen, Germany) were followed by a 2-week differentiation phase. The differentiation medium contained 1.0% DMSO (days 1–3) and later 1.7% DMSO (days 4–14), in addition to the aforementioned supplements (Gripon et al. 2002; Luckert et al. 2018). Treatment of HepaRG cells with cyproconazole or prochloraz was then performed for 24 h in phenol red-free William’s E medium (Pan-Biotech GmbH, Aidenbach, Germany) which contained all ingredients as the differentiation medium including a final DMSO concentration of 1.7%, but only 2% FCS. Afterwards, cells were washed twice with phosphate-buffered saline and were subsequently scraped off the plates in 1 mL lysis buffer. The absence of cytotoxicity of the chosen concentrations had been checked prior to the analyses using the WST-1 cell viability assay in 96-well format according to Knebel et al. (2018b) (data not shown). Experiments were conducted with *n* = 8 (solvent control) or *n* = 4 (treatment) samples per condition.

### Sample preparation

Tissue was homogenized using a ball mill (Micro-dismembrator S, Sartorius, Göttingen). Subsequently, lysis buffer [1% NP-40 (Thermo Scientific, Waltham, USA), 0.01% sodium dodecyl sulfate (Thermo Scientific, Waltham, USA), 0.15 M sodium chloride (Merck, Darmstadt, Germany), 0.01 M di-sodium hydrogen phosphate (Merck, Darmstadt, Germany), 2 mM ethylenediamine-tetraacetic acid (Merck, Darmstadt, Germany) and 2.5 units/mL benzonase (Burlington, Massachusetts, USA)] were added. Primary human hepatocytes were thawed, medium was removed and lysis buffer added to the cell pellet. In case of in vitro samples, cells were washed twice with phosphate-buffered saline and were subsequently scraped off the plates in lysis buffer. All sample types were incubated for 1 h at room temperature under continuous rotation. Debris was precipitated by centrifugation and protein concentration in the supernatant was determined via the Pierce BCA Assay Protein Kit (Thermo Scientific, Waltham, USA). Samples were diluted [50 mM triethanolamine (Carl Roth, Karlsruhe, Germany)], reduced with 5 mM tris(2-carboxyethyl)phosphine (Carl Roth, Karlsruhe, Germany) and denatured for 5 min at 99 °C. Subsequently, the samples were alkylated with 10 mM iodoacetamide (Sigma-Aldrich, Taufkirchen, Germany) and enzymatically digested for 16 h at 37 °C using trypsin (Pierce Trypsin Protease, MS-grade; Thermo Scientific, Waltham, USA) in a 1:20 ratio. The reaction was terminated by heating (5 min at 99 °C) as well as by adding 200 mM phenylmethylsulfonyl fluoride.

### Protein quantification via the TXP methodology

CYP enzyme and transporter expression levels were determined using the previously described TXP methodology (Wegler et al. 2017; Weiss et al. 2018): digest aliquots (10–50 µg protein) were mixed with stable isotope-labeled peptides (customized produced by Intavis, Tübingen, Germany) as internal standards and TXP antibodies (customized produced by Pineda, Berlin, Germany) and incubated for 1 h. The peptide–antibody complexes were precipitated and washed using protein G-coated magnetic beads (Thermo Scientific, Waltham, USA) and an automated magnetic particle processor (King Fisher; Thermo Scientific, Waltham, USA). Peptides were eluted using 1% formic acid (Thermo Scientific, Waltham, USA). Subsequently, the peptides were quantified using the previously described LC–MS methods (UltiMate 3000 RSLCnano and tSIM-QExactive Plus; Thermo Scientific, Waltham, USA) (Weiss et al. 2018). Raw data were processed using Pinpoint 1.4 (Thermo Scientific, Waltham, USA) and the Skyline software (MACOSS Lab, Department of Genome Sciences, University of Washington, Seattle, USA). Peptide amounts were calculated by the peak ratios of the endogenous peptides and the isotope-labeled standards. Mean, standard deviation, coefficient of variation and fold change to control were calculated. In case samples were below the lower limit of quantification (LLOQ), 0.5 LLOQ was used for the calculations**.**

## Results

This study was aimed at comparatively analyzing the hepatic levels of a selection of CYP enzymes and transporters relevant for drug and xenobiotic metabolism between different species, as well as between the in vitro and in vivo situation. For this purpose, the following experimental models were chosen, partially using archived tissue samples from previously published animal studies (Heise et al. 2015; Marx-Stoelting et al. 2017; Rieke et al. 2017; Schmidt et al. 2016): (1) male Wistar rats, (2) male C57/Bl6 wild-type mice, (3) transgenic male CAR/PXR-humanized mice in C57/Bl6 background, (4) transgenic FRG-KO mice with livers repopulated by human hepatocytes, (5) cryopreserved human primary hepatocytes, and (6) differentiated human HepaRG hepatocarcinoma cells. In addition, data from human liver biopsies are presented. This selection covers the full spectrum of experimental systems by comprising classic rodent models, advanced transgenic mouse strains providing different degrees of humanization, and two in vitro models representing the most recognized gold standards for liver metabolism research in cell culture. In a first step, the basal abundance of CYPs and transport proteins was quantified using a targeted proteomics approach and compared to previously published data from human liver biopsies (Weiss et al. 2018). The second part of the study consisted in an analysis of xenobiotic-induced alterations in selected models after exposure to two pesticidal active compounds, cyproconazole and prochloraz (see also the “Introduction” section). The full dataset is available as supplemental files (Supplemental Tables S3–S9). The selection of direct orthologs was possible in some cases (e.g., CYP2E1 present in all species and models analyzed), but not for other CYPs, where no direct orthologs are expressed in the selected species. Here, closely related, well-analyzed and important members of the respective CYP subfamilies were chosen (Table 1; see Supplemental Table S2 for more details).

**Table 1:** Selected orthologous CYP proteins in human, rat and mouse

Human	Rat	Mouse
CYP1A1	CYP1A1	(CYP1A1)
CYP1A2	CYP1A2	(CYP1A2)
CYP2B6	CYP2B1 and CYP2B2	CYP2B10
CYP2C8	CYP2C11	CYP2C29
CYP2C9	CYP2C12	CYP2C38
CYP2C19	CYP2C13	CYP2C39
	CYP2C55	CYP2C55
CYP2D6	CYP2D3	CYP2D9
		CYP2D10
CYP2E1	CYP2E1	CYP2E1
CYP3A4	CYP3A9	
CYP3A5	CYP3A18	CYP3A25
ABCB1	ABCB1a	ABCB1a
ABCB11	ABCB11	ABCB11
ABCC2	ABCC2	ABCC2
ABCC3	ABCC3	ABCC3
SLC10A1	SLC10A1	SLC10A1

Proteins in brackets: not measured in this work (no assays available)

### Inter-species and inter-model comparison of CYP enzyme contents

**Fig. 1:**
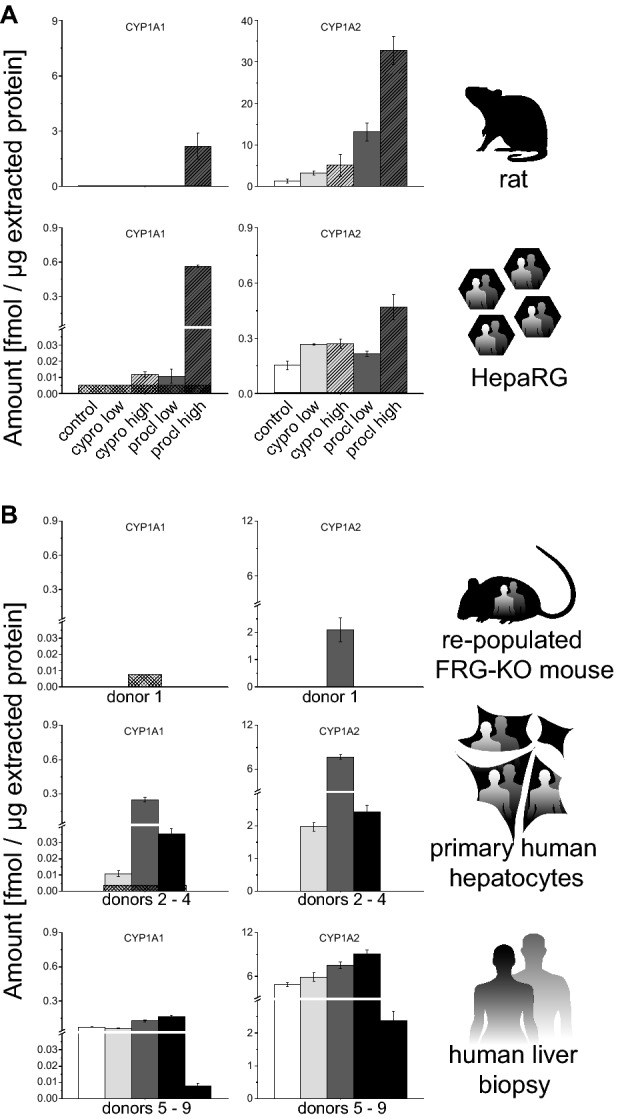
Quantification of CYP1A subfamily enzymes in different model systems. **a** Basal levels as well as induction by cyproconazole and prochloraz were analyzed in the following systems (from top to bottom): rat 28-day study (vehicle control *n* = 10; treatment *n* = 5) and human HepaRG hepatoma cells (solvent control *n* = 8, treatment *n* = 4). No CYP1A assays were available for mouse tissue. For details on dosing and in vitro concentrations, please refer to the “Materials and methods” section. **b** In addition, the basal protein levels were analyzed in following systems: FRG-KO mice repopulated with human hepatocytes (*n* = 8), primary human hepatocytes (three donors shown individually, each measured in three technical replicates), and human liver biopsies (five donors shown individually, each measured in three technical replicates). Mean + SD are shown as absolute values (fmol/µg total protein in the lysate). The squared box indicates 0.5 LLOQ

CYP1A1 and CYP1A2 are present in different species and were thus selected to be monitored as members of the CYP1 subfamily (Fig. 1). As CYP1A TXP assays were only available for rat and human proteins, no data from wild-type or CAR/PXR-humanized mice were available. Rats exhibited very low basal hepatic CYP1A1 levels of about 0.05 fmol/µg protein and substantially higher levels of 1.3 fmol/µg protein CYP1A2. In case of fresh human primary hepatocytes and human liver biopsies, three and five donors were analyzed separately, respectively. As the rats, the human samples contained substantially lower levels of CYP1A1 than CYP1A2 (0.01–0.3 fmol/µg and 2–9 fmol/µg, respectively). Human hepatocytes in vivo in repopulated FRG-KO mice only contained traces of CYP1A1 below the LLOQ, while the CYP1A2 content of 2.1 fmol/µg was almost identical to freshly isolated primary hepatocytes. While similarly containing only traces of CYP1A1, differentiated HepaRG hepatocarcinoma cells contained substantially less CYP1A2 than their primary counterparts did, only 0.15 fmol/µg.

**Fig. 2:**
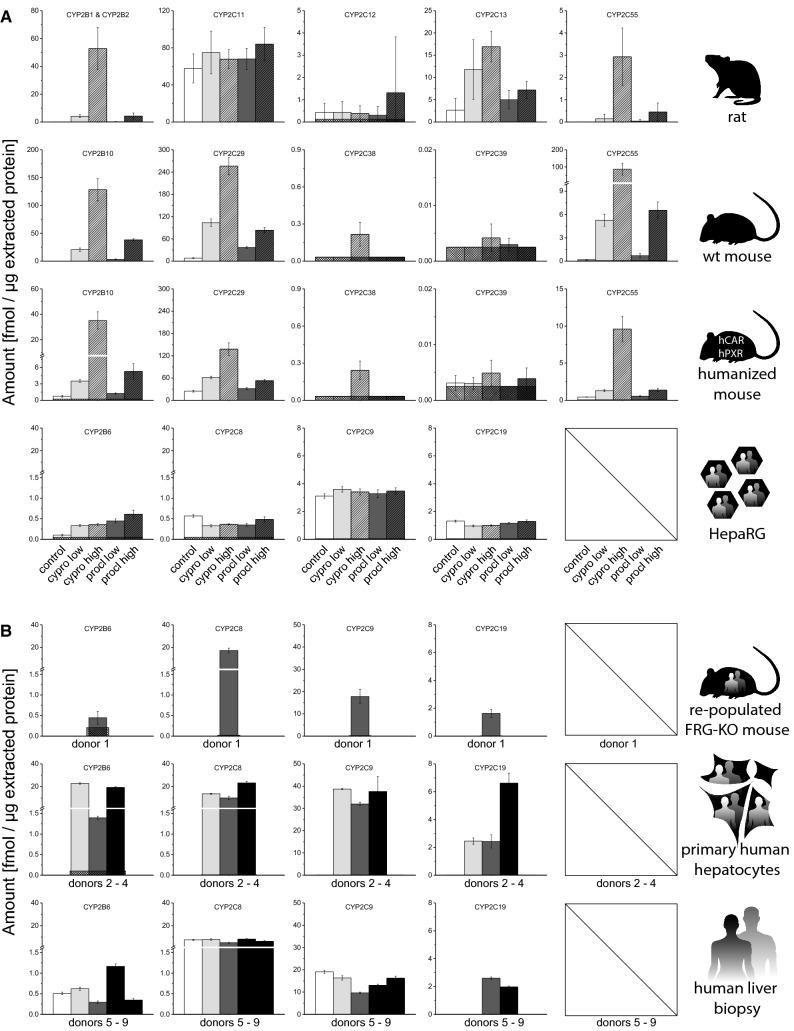
Quantification of CYP2B and CYP2C subfamily enzymes in different model systems. **a** Basal levels as well as induction by cyproconazole and prochloraz were analyzed in the following systems (from top to bottom): rat 28-days study (vehicle control *n* = 10; treatment *n* = 5) and human HepaRG hepatoma cells (solvent control *n* = 8, treatment *n* = 4). For details on dosing and in vitro concentrations, please refer to the “Materials and methods” section. **b** In addition, the basal protein levels were analyzed in following systems: FRG-KO mice repopulated with human hepatocytes (*n* = 8), primary human hepatocytes (three donors shown individually, each measured in three technical replicates), and human liver biopsies (five donors shown individually, each measured in three technical replicates). Mean + SD are shown as absolute values (fmol/µg total protein in the lysate). The squared box indicates 0.5 LLOQ

From the CYP2B subfamily, CYP2B1/2 (rat), CYP2B10 (mouse) and CYP2B6 (human) were analyzed as the most prominent CYP2B members (Fig. 2). A very low basal CYP2B expression was common to most models. CYP2B1/2 in rats, CYP2B10 in wild-type mice and CYP2B6 in HepaRG cells were below 0.2 fmol/µg, while CAR/PXR-humanized mice, human hepatocyte-repopulated FRG-KO mice and human liver biopsies revealed slightly higher CYP2B levels (0.74 fmol/µg CYP2B10, and 0.45 fmol/µg CYP2B6 and 0.3–1.3 fmol/µg, respectively). CYP2B6 content ranged from 1.5 to 23 fmol/µg in primary human hepatocytes.

From the plethora of available CYP2C isoforms in the different species, CYP2C11, CYP2C12, CYP2C13 and CYP2C55 (rat), CYP2C29, CYP2C38, CYP2C39, and CYP2C55 (mouse), and CYP2C8, CYP2C9, and CYP2C19 (human) were chosen (Fig. 2). In rats, the most abundant CYP2C isoform tested was CYP2C11 with 58 fmol/µg, whereas the other isoforms were present at much lower levels (0.01–3 fmol/µg). Mouse CYP2C levels appeared somewhat lower with 8 fmol/µg CYP2C29 and CYP2C38, CYP2C39 as well as CYP2C55 below 0.2 fmol/µg. With humanized CAR and PXR, levels of CYP2C29 were substantially increased to 24 fmol/µg, whereas the levels of CYP2C38, CYP2C39 and CYP2C55 remained low. Among the analyzed CYP2C subfamily members in the human models, CYP2C9 was most abundant and CYP2C19 levels were substantially lower, while the relative CYP2C8 content varied between the models: in human hepatocyte-repopulated livers from FRG-KO mice, CYP2C8 and CYP2C9 were detectable at similar levels (~ 17 fmol/µg), while CYP2C19 was clearly lower at 1.6 fmol/µg. In primary human hepatocytes and liver biopsies, on the other hand, CYP2C8 abundance was in between CYP2C9 and CYP2C19 (10–23 fmol/µg and 5–8 fmol/µg CYP2C8, 32–49 fmol/µg and 9–19 fmol/µg CYP2C9, as well as 2.5–7 fmol/µg and < 3 fmol/µg CYP2C19, respectively). In contrast, the CYP2C content was substantially lower in HepaRG than in the other human models (3 fmol/µg CYP2C9). In addition, it was the only model, in which CYP2C8 abundance was lower than CYP2C19 (0.6 and 1.3 fmol/µg, respectively).

**Fig. 3:**
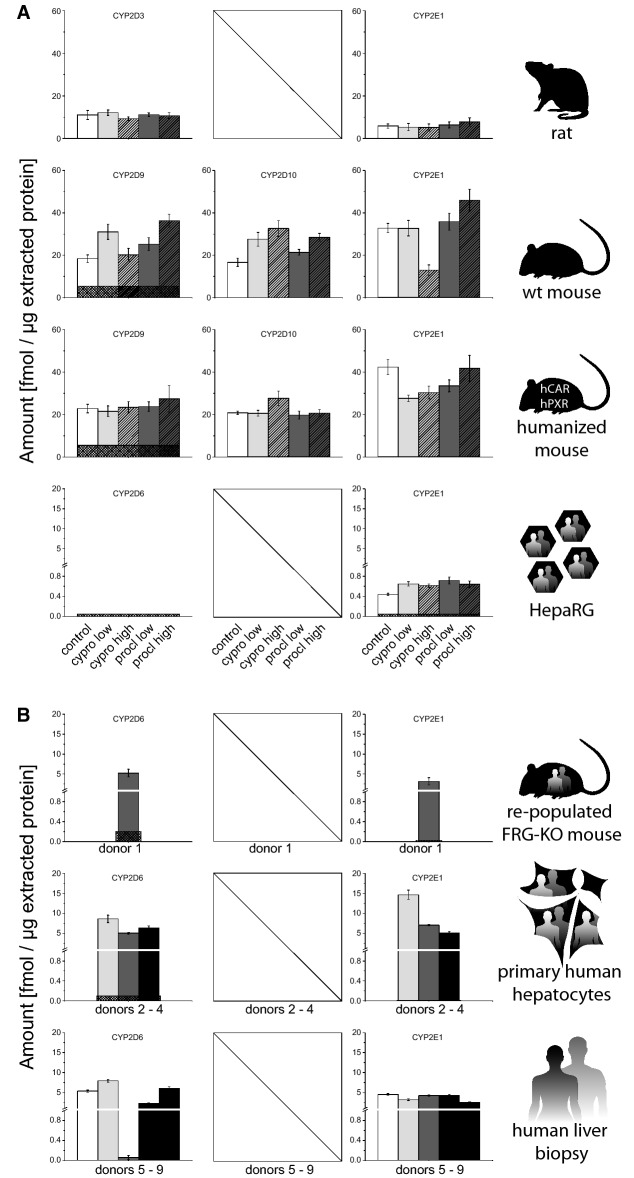
Quantification of CYP2D and CYP2E subfamily enzymes in different model systems. **a** Basal levels as well as induction by cyproconazole and prochloraz were analyzed in the following systems (from top to bottom): rat 28-days study (vehicle control *n* = 10; treatment *n* = 5) and human HepaRG hepatoma cells (solvent control *n* = 8, treatment *n* = 4). For details on dosing and in vitro concentrations, please refer to the “Materials and methods” section. **b** In addition, the basal protein levels were analyzed in following systems: FRG-KO mice repopulated with human hepatocytes (*n* = 8), primary human hepatocytes (three donors shown individually, each measured in three technical replicates), and human liver biopsies (five donors shown individually, each measured in three technical replicates). Mean + SD are shown as absolute values (fmol/µg total protein in the lysate). The squared box indicates 0.5 LLOQ

From the CYP2D subfamily, CYP2D3 (rat), CYP2D9 and CYP2D10 (mouse), as well as CYP2D6 (human) were studied (Fig. 3). Rat CYP2D3 levels were at 11.1 fmol/µg. Levels of CYP2D9 and CYP2D10 were similar in wild-type mice, and there was no substantial difference to mice expressing human CAR and PXR (17–23 fmol/µg). Human primary hepatocytes and liver biopsies displayed levels of 5–9 fmol/µg and 0.6–8 fmol/µg CYP2D6, respectively, which was comparable to the 5.25 fmol/µg of the enzyme observed in human hepatocyte-repopulated FRG-KO mice. CYP2D6 was below the LLOQ in HepaRG cells.

CYP2E1 as the only member of the CYP2E subfamily and expressed in all species and models was also analyzed (Fig. 3). We observed abundant expression in rats (5.9 fmol/µg), wild-type mice (33 fmol/µg), CAR/PXR-humanized mice (42 fmol/µg), repopulated livers of FRG-KO mice (3.2 fmol/µg), primary human hepatocytes (5–15 fmol/µg) and human liver biopsies (2.6–4.6 fmol/µg). Levels of human CYP2E1 in HepaRG cells were the lowest (0.44 fmol/µg).

**Fig. 4:**
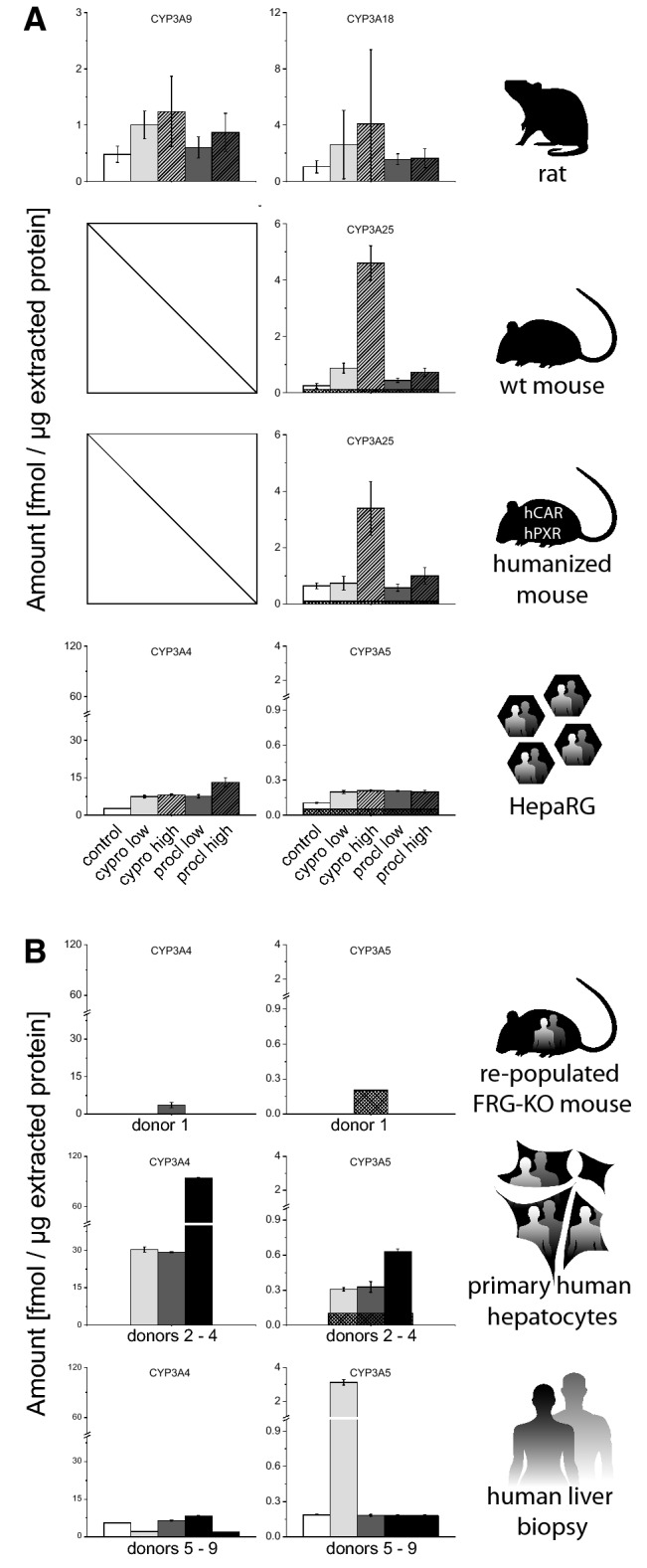
Quantification of CYP3A subfamily enzymes in different model systems. **a** Basal levels as well as induction by cyproconazole and prochloraz were analyzed in the following systems (from top to bottom): rat 28-days study (vehicle control *n* = 10; treatment *n* = 5) and human HepaRG hepatoma cells (solvent control *n* = 8, treatment *n* = 4). For details on dosing and in vitro concentrations, please refer to the “Materials and methods” section. **b** FRG-KO mice repopulated with human hepatocytes (*n* = 8), primary human hepatocytes (three donors shown individually, each measured in three technical replicates), and human liver biopsies (five donors shown individually, each measured in three technical replicates). Mean + SD are shown as absolute values (fmol/µg total protein in the lysate). The squared box indicates 0.5 LLOQ

Finally, expression of the CYP3A subfamily members CYP3A9 and CYP3A18 (rat), CYP3A25 (mouse), and CYP3A4 and CYP3A5 (human) were assessed (Fig. 4). The levels of CYP3A enzymes were rather moderate in rats (< 1 fmol/µg). Wild-type mice and mice with expression of human CAR and PXR displayed a similar low expression of CYP3A25. By contrast, CYP3A subfamily member expression in primary human hepatocytes was 30–90 fmol/µg CYP3A4 and 0.3–0.6 fmol/µg CYP3A5, whereas the abundance of CYP3A4 was substantially lower in liver biopsies (2–7.9 fmol/µg) and comparable in case of CYP3A5 (0.2–3 fmol/µg). The abundance of both proteins was substantially lower in human hepatocyte-repopulated FRG-KO livers, which displayed only 3.6 fmol/µg CYP3A4 along with CYP3A5 levels below the LLOQ. HepaRG cells showed CYP3A expression comparable to the aforementioned liver biopsies.

### Inter-species and inter-model comparison of transport protein contents

**Fig. 5:**
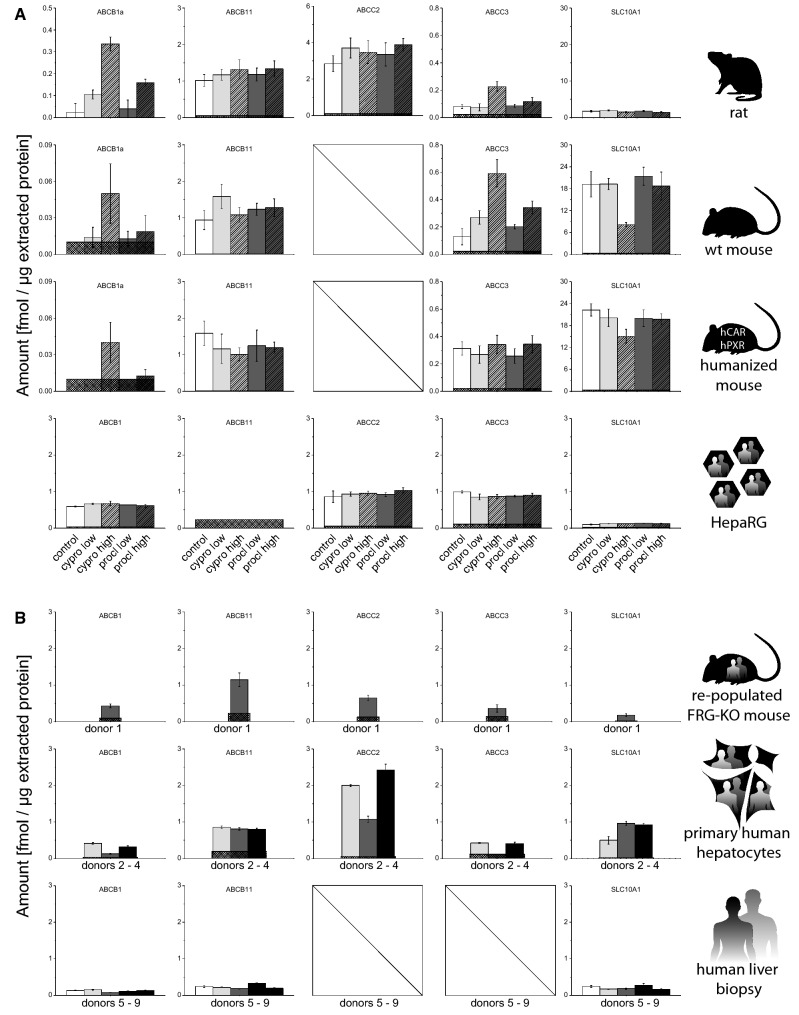
Quantification of selected ABC transporters and SLC10A1 in different model systems. **a** Basal levels as well as induction by cyproconazole and prochloraz were analyzed in the following systems (from top to bottom): rat 28-days study (vehicle control *n* = 10; treatment *n* = 5) and human HepaRG hepatoma cells (solvent control *n* = 8, treatment *n* = 4). For details on dosing and in vitro concentrations, please refer to the “Materials and methods” section. **b** In addition, the basal protein levels were analyzed in following systems: FRG-KO mice repopulated with human hepatocytes (*n* = 8), primary human hepatocytes (three donors shown individually, each measured in three technical replicates), and human liver biopsies (five donors shown individually, each measured in three technical replicates).Mean + SD are shown as absolute values (fmol/µg total protein in the lysate). The squared box indicates 0.5 LLOQ

Four ABC family transporters [ABCB1, also known as multi-drug resistance protein (MDR1); ABCB1a measured in rats and mice, ABCB1 measured in human cells], ABCB11 (bile salt export pump, BSEP), ABCC2 (multi-drug resistance-associated protein 2, MRP2), ABCC3 (multi-drug resistance-associated protein 3, MRP3) and the SLC class transporter SLC10A1 (Na^+^/taurocholate co-transporting peptide, NTCP) were selected for comparative analysis (Fig. 5). With the exception of ABCC2 and ABCC3, all proteins could be assessed in all species and models of interest. In case of ABCC2, no mouse assay was available, whereas ABCC2 and ABCC3 were not part of the previously published study analyzing liver biopsies.

ABCB1a levels were low in rats, in wild-type mice, as well as in mice with humanized CAR and PXR (< 0.02 fmol/µg). In the different human systems, levels were comparable in primary human hepatocytes (0.1–0.4 fmol/µg), liver biopsies (0.08–0.16 fmol/µg), the humanized FRG-KO livers (0.4 fmol/µg) and in HepaRG cells (0.59 fmol/µg). ABCB11 levels between 0.8 and 1.5 fmol/µg were common to most models. By contrast, the level of ABCB11 protein was between 0.2 and 0.3 fmol/µg and below the LLOQ in liver biopsies and HepaRG cells, respectively. A quite similar picture was obtained for ABCC2, where the level of 2.8 fmol/µg in rats was not much different from 1.1 to 2.4 fmol/µg in primary hepatocytes. In case of human hepatocyte-repopulated FRG-KO mice and HepaRG cells, the expression was somewhat lower (0.65 fmol/µg and 0.86 fmol/µg, respectively). In case of ABCC3, the rodent models showed the lowest protein abundance (0.08 fmol/µg in rats and 0.13 fmol/µg wild-type mice). Protein abundance was approximately threefold higher in livers of CAR/PXR-humanized mice and FRG-KO mice (0.31 fmol/µg and 0.36 fmol/µg, respectively). ABCC3 levels in primary human hepatocytes varied fourfold (0.1–0.42 fmol/µg) and were thus in the range of the other models. By contrast, HepaRG cells possessed 1 fmol/µg of ABCC3 protein.

The solute carrier SLC10A1 was present at the highest levels in the mouse models: 19 fmol/µg in wild-type mice and 22 fmol/µg in mice with humanized CAR and PXR. In rats, a medium protein abundance of 1.7 fmol/µg was detected. Expression in the different human models was much lower: The level of 0.5–0.9 fmol/µg SLC10A1 was detected in primary human hepatocytes, while 0.16–0.27 fmol/µg and 0.18 fmol/µg were detected in liver biopsies and human hepatocyte-repopulated livers, respectively. HepaRG cells possessed the lowest levels of the transporter, namely 0.10 fmol/µg. Please note that the SLC10A1 assay simultaneously detects both, human and mouse SLC10A1 in the human hepatocyte-repopulated FRG-KO mouse model and thus detects a sum signal of SLC10A1 from both species; this underlines the high repopulation rate with human hepatocytes in the FRG-KO model (0.18 fmol/µg vs. 19.21 fmol/µg in wild-type mice).

### Fungicide effects on CYP enzyme contents in different model systems

For some, but not all of the model systems, samples treated with the two pesticidal active compounds cyproconazole and prochloraz at two different dose levels were available. Both affect the expression of drug-metabolizing enzymes by activating nuclear receptors (Marx-Stoelting et al. 2020). Cyproconazole preferentially activates CAR and PXR, while prochloraz also shows pronounced activity towards the AHR. The available samples allowed for a comparative analysis of CYP and transporter induction in rats, wild-type mice, mice expressing human CAR and PXR, as well as in HepaRG cells. The doses used in the in vivo experiments were equivalent to the NOAEL and the tenfold of the NOAEL (see also Supplemental Table S1). In vitro concentrations were chosen to reflect the levels of the two compounds reached in the liver after in vivo administration of the above doses as described elsewhere (Seeger et al. 2019).

Cyproconazole did not affect CYP1A1, and increased CYP1A2 protein levels by 2.52- and 3.91-fold in rats after administration of the low and high dose of the compound, respectively (Fig. 1). In HepaRG cells, cyproconazole caused a 2.34-fold increase of CYP1A1 with the high compound concentration, while CYP1A2 induction was 1.74- and 1.77-fold with the low and the high concentration of the compound, respectively. Prochloraz-induced CYP1A1 in rats by 49-fold at the higher, 10 × NOAEL concentration, while CYP1A2 increases were 10- and 25-fold at the two dose levels, respectively. HepaRG cells responded to the lower prochloraz concentration with a 2.15-fold increase of CYP1A1 and a 1.41-fold increase of CYP1A2, whereas the higher concentration led to a 112-fold increase of CYP1A1 and a 3.07-fold increase of CYP1A2. Despite the high fold changes in HepaRG cells, it needs to be noted that the maximum levels of the enzymes were still much lower as compared to rat liver (2.18 fmol/µg CYP1A1 and 33 fmol/µg CYP1A2 in rats vs. 0.56 fmol/µg CYP1A1 and 0.47 fmol/µg CYP1A2 in HepaRG).

In case of wild-type rats, CYP2B1/2 levels were dramatically elevated by cyproconazole (44- and 548-fold at the low and high dose, respectively), while the effect of prochloraz was less pronounced (2.26- and 44-fold induction) (Fig. 2). In wild-type mice, the fold changes were 97- and 608-fold for CYP2B10 with the two cyproconazole doses. Prochloraz provoked 15- and 180-fold increases of CYP2B10. CAR/PXR-humanized mice displayed a 4.7- and 47-fold induction of CYP2B10 by cyproconazole, and 1.7- and 7.1-fold induction of CYP2B10 by prochloraz. It should be noted that the comparison of fold changes alone might be somewhat misleading in case the basal levels of the enzyme of interest are at greater variance between the models, as for example the different CYP2B10 values in the wild-type and humanized mice. Nonetheless, the notable differences in the fold changes are congruent to differences in the total CYP2B10 protein levels, which reach 128 fmol/µg CYP2B10 in wild-type mice with the high dose of cyproconazole, but only 35 fmol/µg in the humanized mouse strain treated with identical doses of the fungicide. CYP2B6 induction in HepaRG cells was similar with both compounds, namely 3.3- and 3.5-fold with cyproconazole and 4.4- and 6-fold with prochloraz. The maximum level of 0.61 fmol/µg CYP2B6 in HepaRG cells, obtained after treatment with the higher concentration of prochloraz, was about two orders of magnitude lower than the maximum CYP2B1/2 levels in rats (52 fmol/µg after high-dose cyproconazole treatment) and wild-type mice (128 fmol/µg, also after high-dose cyproconazole treatment).

Direct comparison of treatment effects on CYP2C isoforms is rather complicated due to the variety of different isoforms in the different species (Fig. 2). In rats, the levels of CYP2C11, the by far most abundant CYP2C isoform tested in this species, raised only moderately after treatment with the two fungicides (maximum 1.5-fold with the high dose of prochloraz). The only effect on CYP2C12 induction was observed with the high dose of prochloraz (3-fold); however, this effect appears questionable because of the high standard deviation of the value. CYP2C13 was induced by both compounds, with cyproconazole (4.4-fold and 6.4-fold) exerting more pronounced effects than prochloraz (1.9- and 2.7-fold with the low and high dose, respectively). High numbers for fold induction were recorded for CYP2C55 with both test compounds (up to 586-fold with the high dose of cyproconazole). This high fold change values were primarily based on the very low basal CYP2C55 content in rat liver. In wild-type mice, the very low levels of CYP2C39 seemed to increase minimally with high-dose cyproconazole treatment, similar to the observation in CAR/PXR-humanized mice. The also low level-expressed CYP2C38 was 6.68-fold induced by high-dose cyproconazole treatment in wild-type mice, comparable to the 7.43-fold induction in mice expressing human CAR and PXR. Abundance of CYP2C55, expressed also at rather low basal levels in mice similar to rats, increased substantially after treatment of wild-type mice with the two dose levels of cyproconazole (29- and 469-fold) and somewhat less pronounced after prochloraz treatment (3.8- and 35-fold). Here, the values for CAR/PXR-humanized mice were considerably smaller with 3- and 22-fold increases caused by cyproconazole and 1.3- and 3.2-fold increases caused by prochloraz. Accordingly, the maximum CYP2C55 levels in mice treated with the higher dose of cyproconazole were clearly different between the wild-type (86.6 fmol/µg) and CAR/PXR-humanized (9.6 fmol/µg) mice. The most abundant of the analyzed CYP2C isoforms in wild-type mice, CYP2C29, increased after treatment with cyproconazole (12.8-fold and 32-fold) as well as with prochloraz (4.6- and tenfold, respectively). These values were higher than in CAR/PXR-humanized mice (2.5- and 5.7-fold for cyproconazole and 1.3- and 2.2-fold for prochloraz, respectively). This discrepancy is based on two findings: first, the basal expression of CYP2C29 was substantially higher in mice with human CAR and PXR, as compared to wild-type mice (24 fmol/µg vs. 8 fmol/µg). Second, the maximum CYP2C29 levels reached after induction were substantially higher in wild-type mice, as compared to the humanized group (256 fmol/µg vs. 138 fmol/µg with the high dose of cyproconazole, respectively). In HepaRG cells, no induction of CYP2C8, CYP2C9 or CYP2C19 was observable.

CYP2D3 expression did not change upon cyproconazole or prochloraz treatment (Fig. 3). Mouse CYP2D9 and CYP2D10 protein levels also remained rather unchanged by the two fungicides in mice with human CAR and PXR, whereas a modest increase (up to 1.98-fold after treatment with the high dose of prochloraz) was visible in wild-type mice. CYP2D6 expression, undetectable in HepaRG cells at basal levels, was also not detectable after treatment of cell cultures with cyproconazole or prochloraz. Effects of treatment on CYP2E1 levels could be analyzed in models representing all three species (Fig. 3). The levels of the protein did not change in a notable manner in rats. In wild-type mice, the expression of CYP2E1 dropped to 40% of the initial level after treatment with the higher dose of cyproconazole, whereas the other treatments did not exert pronounced effects. CYP2E1 levels in CAR/PXR-humanized mice also decreased slightly following administration of cyproconazole (by 35% and 28%, respectively, at the low and high doses), but the effect was less pronounced and not dose-dependent. In HepaRG cells, CYP2E1 levels increased slightly with both treatments, but again without a clear dose-dependency.

Finally, levels of CYP3A isoforms were determined (Fig. 4). Rat CYP3A9 was moderately increased following treatment of animals with both compounds (2- and 2.6-fold with cyproconazole, 1.3- and 1.8-fold with prochloraz, at the low and high doses, respectively). This was similar to the observed induction of CYP3A18 (2.5- and 3.9-fold with cyproconazole, 1.5- and 1.6-fold with prochloraz). CYP3A25 expression in wild-type mice strongly rose following cyproconazole treatment (3.7- and 19-fold), while the effects obtained with prochloraz were much weaker (1.8-fold and threefold). In mice with human CAR and PXR, the lower doses of both test compounds did not result in clear CYP3A enzyme induction, while the higher doses induced CYP3A25 by 5.3-fold (cyproconazole) and more moderately by 1.6-fold (prochloraz). The maximum induced values of the CYP3A isoforms were in a comparable range in all three rodent models (rat: 1.2 fmol/µg for CYP3A9 and 4 fmol/µg for CYP3A18; wild-type mouse: 4.6 fmol/µg for CYP3A25; CAR/PXR-humanized mouse: 3.4 fmol/µg for CYP3A25; all measured after high-dose cyproconazole treatment). The higher fold change values calculated for the receptor-humanized mouse model mainly result from the different basal CYP3A25 levels. In addition, in HepaRG cells, CYP3A induction was clearly visible: treatment with cyproconazole led to a 2.8-fold and 3-fold induction of CYP3A4 at the low and high concentration, while treatment with prochloraz was able to induce CYP3A4 protein levels by 2.8- and 4.9-fold at the respective concentrations.

### Fungicide effects on transport protein contents in different model systems

Cyproconazole and prochloraz effects on different transport proteins were also studied (Fig. 5). Rat ABCB11, ABCC2 and SLC10A1 were not responsive to the treatments in a notable manner. By contrast, rat ABCB1a was increased by cyproconazole (4.3- and 13.7-fold) and also by prochloraz (1.6- and 6.5-fold, respectively, with the low and high doses of the two compounds). ABCC3 induction was only seen after administration of high-dose cyproconazole (2.8-fold) or prochloraz (1.5-fold). In wild-type mice, ABCB11 expression was not substantially altered by the treatments. In addition, similar to the situation in rats, ABCB1a was inducible by high doses of cyproconazole (fivefold) and prochloraz (1.9-fold). Comparable values were obtained for ABCC3 with maximum inductions of 4.6-fold (high-dose cyproconazole) and 2.7-fold (high-dose prochloraz). The expression of SLC10A1 was not changed with the exception of high-dose cyproconazole treatment, where a 58% loss of protein abundance was observed, comparable to the above-mentioned reduction of CYP2E1 levels. Mice with human CAR and PXR did not show remarkably increased ABCB11, ABCC3 and SLC10A1 levels. The decrease of SLC10A1 protein levels at high-dose cyproconazole was also visible in this mouse model, even though to a somewhat lesser extent than in wild-type mice (32% decrease vs. 58% decrease). ABCB1a was clearly affected by high-dose cyproconazole (4-fold increase) in CAR/PXR-humanized mice, whereas the effect of high-dose prochloraz was almost negligible (1.2-fold increase). No notable changes were observed for the analyzed ABC transporters ABCB1, ABCC2 and ABCC3, as well as for SLC10A1 in HepaRG cells.

## Discussion

In this paper, we present a comprehensive and comparative cross-species overview of the expression of important drug-metabolizing CYP enzymes from the CYP families 1–3, as well as of relevant drug and xenobiotic transporters. There are several in vitro and in vivo models which are used to predict toxicity and pharmacokinetics in man. Cell culture experiments are often used to examine specific aspects, such as protein induction or substrate transport across membranes. For more complex questions, animal models are utilized, of which rat and mouse are most common. In addition, humanized animal models, in which one or more proteins are replaced by the human variants due to genetic modifications, gain importance (Scheer and Wilson 2016; Scheer and Wolf 2014; Xie and Evans 2002). Recently, a mouse model has been published in which 33 murine CYPs as well as the receptors CAR and PXR were substituted by the human receptors and CYPs (Henderson et al. 2019). A great challenge of these studies is to compare the results of the different models and to draw meaningful conclusions for pharmacokinetics or the toxicological outcome in humans (Bogaards et al. 2000). CYP enzymes are classified according to their homology, and their expression levels and isoforms vary strongly between species. At the enzyme activity level, one amino acid exchange may alter the substrate specificity of the enzyme (Martignoni et al. 2006), further impeding direct comparability between the different species with their highly homologous but nonetheless often slightly different CYP enzymes or transport proteins. To the best of our knowledge, this is the first paper to compare the levels of individual CYP enzymes and transporters in a selection of experimental models ranging from classic laboratory rodents to advanced transgenic animals and gold-standard human liver primary cells and biopsies.

Antibody-based separation and quantification of CYPs and transport proteins, especially across species, by classic approaches such as Western blotting is difficult or even impossible, for example as a consequence of antibody cross-reactivity with closely related isoforms, or due to a lack of appropriate external standards. The quantitative comparison of the inductive potential of foreign substances is made easier by TXP assays, which can be applied for several species. They are based on the quantification of proteotypic peptides, which can be assigned uniquely to one protein in the species of interest. These peptides are enriched by TXP antibodies, which recognize four C-terminal amino acids. If the C-terminal sequence is conserved, the same antibody can be used to for several closely related isoforms (Planatscher et al. 2010; Weiss et al. 2015). This was for example the case for CYP2B6, CYP2C8 and CYP2E1 in human. In addition, the sequence was also conserved across species and the antibody could also be used to analyze four CYP2C isoforms in mouse, as well as CYP2B10 and CYP2C55 in rat. In case of CYP2C55, as well as CYP2E1, ABCB1a and ABCC3, not only the C-terminal sequence, but also the total peptide sequence is conserved between mice and rats allowing quantification across species with the same assay.

It should be noted that some facts complicate direct comparisons between species and models. First, only for some CYPs direct orthologs are available in all three species studied. We, therefore, chose the approach of presenting data on, e.g., different CYP2C subfamily members from each species. Second, in a complex model such as the FRG-KO mice repopulated with human hepatocytes, individual TXP assays may not work because the used antibody recognizes not only the intended human CYP-derived peptide, but also peptide(s) originating from mouse proteins. Therefore, a very minor fraction of measurements could not deliver conclusive results. Third, the normalization of data is a critical step. We chose to refer to the total protein content of a sample as an objective and easily accessible parameter. Nonetheless, the values derived that way might deviate from the CYP or transporter content per hepatocyte, due to the fact that cell sizes are different among the models and also because in vivo additional protein is present in the analyses from extracellular matrix and non-parenchymal cells. However, the great heterogeneity of cell sizes and CYP content within a single liver [see review papers on hepatic zonation, e.g., Braeuning and Schwarz (2010), Gebhardt (1992), and Oinonen and Lindros (1998)] would make it unreliable to express the measured protein values per cell. Fourth, comparisons including primary human hepatocytes or models built upon these cells are always critical, because there is a great variance between individual donors, especially with respect to drug-metabolizing enzymes (Zanger et al. 2005; Zanger and Schwab 2013). We addressed this issue by analyzing primary hepatocytes from three different donors and presenting additional data on human liver biopsies of five donors (Weiss et al. 2018). Nevertheless, the values derived from biopsies, primary hepatocytes and from FRG-KO mice repopulated with human cells originating from one primary hepatocyte donor, should be considered exemplary values and not fixed numbers representative for primary human hepatocytes from other sources. Due to the above-mentioned variability of human-derived data and because of the difficulty of clearly assigning individual protein homologs between the species, we decided not to perform statistical testing, as the use of doing so would appear, if present at all, very limited.

However, there are several notable findings that are worth being discussed: CYP2B6, for example, was expressed at high levels in human primary hepatocytes, whereas the levels of CYP2B enzymes in all other models were considerably lower. Such findings raise the general question of the transferability of results yielded with the different models or species when a specific enzyme (subfamily) is in the focus of a particular study. More interestingly, CYP2B6 and especially CYP3A4 were highest in primary human hepatocytes, whereas the expression of the same enzymes in human hepatocytes in the livers of repopulated FRG-KO mice and human liver biopsies was much lower, close to the low levels of other CYP2B or CYP3A subfamily members in rats or wild-type mice. For many analytes, considerable differences were visible between HepaRG cells on the one hand, and human liver tissue, primary human hepatocytes and human hepatocytes growing within the FRG-KO livers on the other hand. In general, HepaRG cells contained lower levels of CYP enzymes and transporters than the cryopreserved primary human hepatocytes. The effect was much more pronounced in the case of CYP enzymes. The only exception were ABCB1 and ABCC3, which were expressed at a similar level and 2.3-fold higher in HepaRG than in liver biopsies and primary human hepatocytes, respectively. HepaRG cells are generally regarded to constitute a human hepatocyte model superior to other permanent hepatic cell lines. The present analysis nonetheless revealed also pronounced differences between HepaRG and primary cells, suggesting that HepaRG might not be suited to entirely substitute primary cells with respect to liver metabolism and toxicity studies. Of note, HepaRG cells were used here under conditions of the maximum DMSO concentration of 1.7%. This ensures the highest basal expression of CYPs (Antherieu et al. 2012). On the other hand, the increase in CYP expression caused by DMSO may be an underlying reason for a diminished inducibility of CYPs by xenobiotics as compared to HepaRG cells grown with less DMSO (i.e., smaller fold changes are achieved by xenobiotics due to the already higher basal expression caused by the DMSO). The suitability of HepaRG cells for in vitro studies of human liver metabolism or xenobiotic enzyme induction has been investigated in detail by others; for example see Aninat et al. (2006), Antherieu et al. (2012), or Guillouzo et al. (2007) and also more recent work by Berger et al. (2016), Bernasconi et al. (2019), Kvist et al. (2018), and Yokoyama et al. (2018). The aforementioned studies conclude that HepaRG cells constitute a very good in vitro model for human liver, which closely resembles the in vivo situation and is superior to standard hepatoma cell lines. Nonetheless, as also revealed in this study, there are still certain differences in CYP expression and inducibility between HepaRG cells and primary hepatocytes, which should be taken into account when interpreting in vitro test results.

With respect to CYP and transporter induction, it was beyond the scope of this manuscript to perform induction studies in all species and model systems, as the main focus was set on the analysis of existing samples from animal studies, thus avoiding additional animal experimentation. Hence, we present CYP induction data using xenobiotic inducers for three rodent and one human model systems: (1) male Wistar rats, (2) male C57/Bl6 wild-type mice, (3) transgenic male CAR/PXR-humanized mice in C57/Bl6 background, and (4) differentiated human HepaRG hepatocarcinoma cells. HepaRG cells were chosen for induction studies as this cell line has turned out to be better suited for enzyme induction studies than primary hepatocytes in an extensive validation study (Bernasconi et al. 2019). For CYP1A, the induction data demonstrate that subfamily member inducibility is present in rats in vivo as well as in HepaRG cells in vitro. The stronger activity of prochloraz as compared to cyproconazole reflects the compounds’ nuclear receptor activation profiles. CYP1A induction by cyproconazole and prochloraz has already been reported [for review see Marx-Stoelting et al. (2020)], and general responsiveness to AHR activators has also been demonstrated in HepaRG cells with other agonists of the receptor, e.g., see Knebel et al. (2018a), Tanner et al. (2018), and Thomas et al. (2015).

CYP induction can in general be looked at based on fold change values, or based on absolute quantification of the respective protein. For example, basal as well as prochloraz-induced CYP1A2 abundance is substantially higher than CYP1A1 in rats, which is also in concordance with the literature (Martignoni et al. 2006). Basal CYP1A2 is 28-fold more abundant and after induction by prochloraz it is 15-fold more abundant than CYP1A1. This results in a 2-fold higher fold change of CYP1A1 abundance induced by prochloraz compared to CYP1A2. In contrast to that, basal CYP1A2 is 30-fold higher than CYP1A1 in HepaRG, but equally abundant after prochloraz induction. Taking into account only the fold changes, prochloraz has a greater impact on CYP1A1 than CYP1A2 as well as on human CYP1A1 than rat CYP1A1. Thus, fold induction as well as absolute amounts should to be taken into account for data interpretation when comparing CYP isoforms in one model as well as when comparing a specific CYP between different models. In HepaRG cells, the induced levels of CYP1A2 in the cell line remain below the levels reached in rats in vivo, in terms of fold change as well as in terms of absolute amounts. The absolute protein level values, as well as the fold changes observed with the CYP3A isoforms upon the treatment of animals or cell cultures with cyproconazole or prochloraz show a good correlation between rodents in vivo and human HepaRG cells in vitro.

At the enzyme induction level, also some unexpected differences between the wild-type and the receptor-humanized mice become evident: especially CYP2B9, CYP2B10, CYP2C29 and CYP2C55 responded with less induction in CAR/PXR-humanized mice, as compared to their wild-type counterparts. This may indicate that the human receptors CAR and PXR are less powerful activators of target gene expression than their murine counterparts are. This is in line with data on mRNA induction of *Cyp2b10* gene expression by prochloraz and cyproconazole also showing more pronounced effects in wild-type mice (Marx-Stoelting et al. 2017). Putative different activities of receptors in individual species may be of relevance also for endpoints other than CYP induction: for example, human relevance of the non-genotoxic, CAR-mediated mechanism for liver tumor induction is discussed controversially (Braeuning 2014; Braeuning and Schwarz 2016; Elcombe et al. 2014). Besides potential qualitative differences between rodents and humans, also quantitative differences should be considered when assessing human health risks resulting from CAR-activating chemicals. With respect to studies of the metabolism of foreign compounds, the pronounced differences in the induction of several CAR and/or PXR target genes between wild-type and CAR/PXR-humanized mice might complicate the interpretation of results, if the compound of interest is an inducer and substrate of the aforementioned CYP enzymes.

Another interesting observation is the strong downregulation of CYP2E1 in wild-type mice by cyproconazole. Such a phenomenon was not observed in rats or in human HepaRG cells in our study, and CAR/PXR-humanized mice showed this effect to a lesser degree. One might speculate that the phenomenon is related to the onset of hepatotoxicity and regenerative proliferation which was observed in wild-type but not humanized mice in the original study (Marx-Stoelting et al. 2017). In principle, a cytotoxicity-dependent loss of perivenous hepatocytes, which express the highest levels of most CYPs including CYP2E1, may explain the decrease in CYP2E1 levels. Preferential perivenous destruction of CYP-expressing hepatocytes is frequently seen with hepatotoxic compounds, for example see Hessel-Pras et al. (2020), Hohme et al. (2007), Schenk et al. (2017), and Sekine et al. (2006). CYP2E1 expression is, contrasting the situation with other CYPs, barely regulated at the mRNA level. Thus, the loss of some of the CYP2E1-expressing cells may explain the loss of the protein, while the induction of other CYPs in the remaining surviving cells could mask the loss of other CYPs. However, besides consequences of general perivenous toxicity, it appears also possible that an unknown, more specific mechanism downregulates CYP2E1 in murine hepatocytes upon treatment with cyproconazole. In any case, the results are in line with Martignoni et al. (2006) suggesting that rats may be the best animal model with respect to predicting CYP2E1 effects in human. In addition, results obtained with the humanized mouse model were more in line with other models than the wild-type mouse model suggesting that humanized animal models can be helpful to extrapolate effects in man from experimental animal data.

In summary, the present study provides a broad-spectrum overview of CYP enzyme and transport protein contents of well-established and state-of-the-art models for liver toxicity and pharmaco-/toxicokinetics. The data can help with the appropriate design of new studies and support the interpretation of data from the analyzed models with respect to inter-model transferability of results and human relevance. Finally, the TXP technology has proven its suitability for the use as a powerful tool in cross-species protein characterization.

## Electronic supplementary material

Below is the link to the electronic supplementary material.

Supplementary file1 (DOCX 14 kb)

Supplementary file2 (XSLX 68 kb)

## Data Availability

Datasets are available as supplemental files. Raw datasets are available from the corresponding author on reasonable request.

## Abbreviations

ABC: ATP-binding cassette
AHR: Aryl hydrocarbon receptor
CAR: Constitutive androstane receptor
CYP: Cytochrome P450
DMSO: Dimethyl sulfoxide
FCS: Fetal calf serum
LLOQ: Lower limit of quantification
MDR: Multi-drug resistance protein
MRP: Multi-drug resistance-associated protein
NOAEL: No observed adverse effect level
NTCP: Na^+^/taurocholate co-transporting peptide
PXR: Pregnane-X-receptor
SLC: Solute carrier
TXP: Triple-X-proteomics
