# Supplementary material for: Cross-species analysis of hepatic cytochrome P450 and transport protein expression
Source: Arch Toxicol. 2020 Nov 4;95(1):117–33. doi: 10.1007/s00204-020-02939-4 (PMC7811513; doi:10.1007/s00204-020-02939-4)
Supplement: Supplementary file 1 — Supplementary file1 (DOCX 14 kb) [file 204_2020_2939_MOESM1_ESM.docx]

Supplemental table 1: Treatment of rodent and invitro models. The tested model systems were treated with cyproconazole and prochloraz. For experimental details, please refer to the method section and the following publications: (Heise et al. 2015; Marx-Stoelting et al. 2017; Schmidt et al. 2016).

Supplemental table 2: Details of the analyzed proteins. Protein name, acronym, the recommended name, gene name and Uniprot entry ID are listed here.

Supplemental table 3: Overview of surrogate peptides. All covered proteins are listed together with the surrogate peptide used for quantification.

Supplemental table 4: Quantification of CYPs and transporter in male Wistar rats. Mean, SD, CV, and fold change to control are listed as well as 0.5 LLOQ. In case the value was below LLOQ, it was replaced by 0.5 LLOQ calculation of mean, SD, CV and fold change.

Supplemental table 5: Quantification of CYPs and transporter in male C57/Bl6 wildtype mice. Mean, SD, CV, and fold change to control are listed as well as 0.5 LLOQ. In case the value was below LLOQ, it was replaced by 0.5 LLOQ calculation of mean, SD, CV and fold change.

Supplemental table 6: Quantification of CYPs and transporter in transgenic male CAR/PXR-humanized mice in C57/Bl6 background. Mean, SD, CV, and fold change to control are listed as well as 0.5 LLOQ. In case the value was below LLOQ, it was replaced by 0.5 LLOQ calculation of mean, SD, CV and fold change.

Supplemental table 7: Quantification of CYPs and transporter in transgenic FRG-KO mice with livers repopulated by human hepatocytes. Mean, SD, CV, and fold change to control are listed as well as 0.5 LLOQ. In case the value was below LLOQ, it was replaced by 0.5 LLOQ calculation of mean, SD, CV and fold change.

Supplemental table 8: Quantification of CYPs and transporter in cryoconserved human primary hepatocytes. Mean, SD, CV, and fold change to control are listed as well as 0.5 LLOQ. In case the value was below LLOQ, it was replaced by 0.5 LLOQ calculation of mean, SD, CV and fold change.

Supplemental table 9: Quantification of CYPs and transporter in differentiated human HepaRG hepatocarcinoma cells. Mean, SD, CV, and fold change to control are listed as well as 0.5 LLOQ. In case the value was below LLOQ, it was replaced by 0.5 LLOQ calculation of mean, SD, CV and fold change.

Supplemental table 10: Quantification of CYPs and transporter in human liver biopsies. Mean, SD, CV, and fold change to control are listed. This data has already been published (Weiß et al. 2018) and was listed here to make comparison easier.
